# Influence of Contrast and Coherence on the Temporal Dynamics of Binocular Motion Rivalry

**DOI:** 10.1371/journal.pone.0071931

**Published:** 2013-08-14

**Authors:** Artem Platonov, Jeroen Goossens

**Affiliations:** Section Biophysics, Department of Cognitive Neuroscience, Donders Institute for Brain, Cognition and Behaviour, Radboud University Medical Centre Nijmegen, Nijmegen, The Netherlands; University of Muenster, Germany

## Abstract

Levelt’s four propositions (L1–L4), which characterize the relation between changes in “stimulus strength” in the two eyes and percept alternations, are considered benchmark for binocular rivalry models. It was recently demonstrated that adaptation mutual-inhibition models of binocular rivalry capture L4 only in a limited range of input strengths, predicting an increase rather than a decrease in dominance durations with increasing stimulus strength for weak stimuli. This observation challenges the validity of those models, but possibly L4 itself is invalid. So far, L1–L4 have been tested mainly by varying the contrast of static stimuli, but since binocular rivalry breaks down at low contrasts, it has been difficult to study L4. To circumvent this problem, and to test if the recent revision of L2 has more general validity, we studied changes in binocular rivalry evoked by manipulating coherence of oppositely-moving random-dot stimuli in the two eyes, and compared them against the effects of stimulus contrast. Thirteen human observers participated. Both contrast and coherence manipulations in one eye produced robust changes in both eyes; dominance durations of the eye receiving the stronger stimulus increased while those of the other eye decreased, albeit less steeply. This is inconsistent with L2 but supports its revision. When coherence was augmented in both eyes simultaneously, dominance durations first increased at low coherence, and then decreased for further increases in coherence. The same held true for the alternation periods. The initial increase in dominance durations was absent in the contrast experiments, but with coherence manipulations, rivalry could be tested at much lower stimulus strengths. Thus, we found that L4, like L2, is only valid in a limited range of stimulus strengths. Outside that range, the opposite is true. Apparent discrepancies between contrast and coherence experiments could be fully reconciled with adaptation mutual-inhibition models using a simple input transfer-function.

## Introduction

Binocular rivalry is a phenomenon which occurs when our eyes receive stereo-incompatible inputs at the same retinal location. This leads to perceptual alternations between the two images even though both stimuli are constantly present. The majority of current models of binocular rivalry assume that alternations between the two dominance states involve interaction between feedback cross-inhibition and slow self-adaptation [1], [2], [3], [4], [5]. In these models, populations of neurons which represent the competing percepts inhibit each other through their output, resulting in suppression of one percepts and dominance of the other. As a result of adaptation, the inhibition from the dominant population to the suppressed one slowly decays allowing the suppressed population to become dominant, which in turn allows the previously dominant population to recover from adaptation.

Although several studies have challenged the role of adaptation [but see e.g. 6,7,8], there is substantial evidence that adaptation plays a significant role in binocular rivalry alternations [9], [10], [11], [12], [13], [14]. Evidence for the involvement of reciprocal inhibition originates from Levelt’s [15] influential study on binocular rivalry dynamics which characterized how alternations between the two percepts are affected by the strengths of the stimuli in the two eyes. More specifically, Levelt’s second proposition (L2) entails (together with L1 and L3) that if the stimulus strength is changed in one eye, it affects exclusively the dominance durations of the contralateral eye, while having no effect at all on the dominance durations of the eye in which the stimulus strength is manipulated [16]. This result may seem counterintuitive at first glance, but is easily explained within the framework of reciprocal inhibition where a given stimulus generates not an isolated response but one linked to the response generated by another, competing stimulus. L2 was reconfirmed in the way it was stated by Levelt [17], [18], and later in a more attenuated form, which states that there are also changes (albeit much smaller) in dominance durations of the ipsilateral eye [19], [20]. More recently, this view on L2 was challenged by Brascamp et al. [21] who, by testing a wider range of stimulus contrasts, found that dominance durations mainly changed for stimuli in the eye which received the higher-contrast image [see also 22,23]. The fourth proposition (L4) posits that increasing the stimulus strength in both eyes shortens the suppression phases of stimuli in both eyes, leading to increases in the rivalry alternation rate, i.e., decreases in dominance duration of the two competing percepts.

Levelt’s four propositions became an experimental hallmark for testing theoretical models of binocular rivalry, and one appeal of adaptation mutual-inhibition models is their ability to successfully replicate them [24], [25]. However, it was recently demonstrated that most of these models capture L4 only in a limited range of stimulus strengths, because they predict an increase in percept durations as a function of the increasing input strength for a range of low input values [26], [27]. This behavior is often considered as a limitation of adaptation mutual-inhibition models to explain binocular rivalry [6], [28]. But, the apparent mismatch between empirical data and the model prediction may also be due to the limitations imposed by the inability of low contrast stimuli to evoke binocular rivalry [29].

In general, however, contrast is not the only physical parameter that determines ‘stimulus strength’. For example, in a wide range of perceptual studies, the percentage of coherently moving dots is used to manipulate the strength of random-dot motion stimuli [e.g. 30,31,32,33,34,35]. Moreover, preliminary data indicates that two rivalry motion stimuli with coherence values near the motion discrimination threshold still elicit perceptual alternation [36]. This suggests that coherence could be a better candidate for testing L4 at low stimulus strengths.

The possibility exists, however, that motion coherence does not alter the average signal amplitude. Instead, changes in coherence could influence the noise within the competing populations. If so, one should expect that the effect of manipulating the percentage of coherently moving dots differs from the effects of changing stimulus contrast, and that it can be described by changing the amount of noise on the input in adaptation mutual-inhibition models.

So far, experimental tests of Levelt’s propositions have primarily focused on contrast manipulations [e.g. 17,20,21], but to our knowledge, they have not yet been tested for coherence. We thus studied changes in dominance alternations evoked by manipulating either contrast or coherence of rivalrous motion stimuli. The results were compared against the predictions from an adaptation mutual-inhibition model (Noest et al., 2007) in which we changed either the amplitudes or the amount of noise on the inputs.

Most of the studies testing the validity of Levelt’s propositions have used a forced-choice paradigm in which subjects always had to choose between the two competing stimuli. However, by manipulating the stimulus strength one might also influence the occurrence of non-exclusive percepts, such as piecemeal and transparent percepts (i.e., mixed percepts). For very faint stimuli one might even expect that subjects are no longer aware of them or, if they are too noisy, that they do not elicit any coherent percept whatsoever (i.e., ‘null’ percepts). In this study, we therefore asked our subjects to also indicate mixed and ‘null’ percepts so that we could dissociate them from exclusive dominance states.

## Methods

### Subjects and Setup

Thirteen human volunteers with normal or corrected to normal visual acuity participated in this study. Some of the subjects participated in previous binocular rivalry experiments, but all subjects were kept naïve regarding the purpose of the present study. The subjects gave informed consent in writing prior to their participation. The experiments were approved by the Radboud University Nijmegen Medical Centre.

Subjects were seated in front of an LCD computer screen (ViewSonic, VX1940w) in an otherwise dark room. Their head and chin was supported by a forehead rest and chin cup. The visual stimuli were generated with a personal computer equipped with an openGL graphics card and presented to the subjects’ left and right eye by means of a front-mirror stereoscope (HyperView, Berezin, U.S.A.). The total viewing distance was 67 cm. The screen resolution was 1680×1050 pixels with an image refresh rate of 60 Hz. We used a precision Minolta Luminance Meter LS-100 to calibrate the display.

Subjects indicated their percepts by pressing mouse buttons. Button states were recorded by the stimulus program and stored for offline analysis.

### Visual Stimuli

Stimuli were generated with Matlab (The MathWorks, Inc.) using the Psychophysics Toolbox extensions [37], [38]. The visual motion stimuli used in the motion rivalry experiments consisted of two independently generated random dot kinematograms (RDKs). Each RDK consisted of 533 white dots (2×2 pixels, 0.05°) that moved against the background within a 4° circular aperture. Every signal dot started at a random location within the aperture and then moved at 4.2°/s for a fixed duration of 4 frames, except in the beginning. In the beginning, each signal dot was assigned a random lifetime between 1 and 4 frames so that, on average, ¼ of the signal dots were replaced with a new set of dots on each subsequent frame of the sequence. If a signal dot reached the boundary of the aperture before its lifetime expired, it was wrapped to the opposite side. Noise dots – if present - were displaced to a new random location within the aperture on each subsequent frame of the sequence. We manipulated either the contrast or the percentage of coherently moving dots, keeping the other parameter fixed at the highest possible value. This ensured that stimulus strength in the other physical stimulus dimension would not hamper the competition.

### Experimental Procedures

#### Motion discrimination thresholds

For all participants, we first determined their motion discrimination threshold for stimulus contrast and motion coherence, respectively. Towards that end, we used a two-alternative forced-choice (2AFC) motion-direction discrimination task in which identical, unambiguous stimuli were presented to the two eyes. Each trial started with a button press, followed by the presentation of a central fixation point which the subject had to fixate for the remainder of the trial. Subsequently, a visual motion stimulus was presented for 0.5 seconds. At the end of each stimulus presentation, the subject had to indicate the perceived direction of motion, guessing if necessary. In two separate sessions, of 600 trials each, either stimulus contrast, or motion coherence was manipulated in a pseudorandom fashion.

To determine the thresholds for contrast, we presented 100% coherently moving signal dots against a gray background (15 cd/m^2^), and we set the luminance of all dots to one of four possible levels between 15.8 cd/m^2^ and 19.5 cd/m^2^. This resulted in a contrast range of 2.5–13% Michelson. To measure the thresholds for coherence, we presented equiluminant white dots (14 cd/m^2^) moving against a black background (0.12 cd/m^2^), and we manipulated the percentage of coherently moving dots between 1% and 25% (six levels).

From the resulting psychometric response curves, we determined the 75%-correct discrimination thresholds. These threshold values were then averaged across subjects and the resulting means were used to calculate matching stimulus contrast and coherence values for the binocular rivalry experiments. The average threshold for stimulus contrast was 6.4±0.6% Michelson (mean±SD, with stimulus coherence fixed at 100%). For motion coherence, the averaged threshold was 4.0±0.4% coherence (with stimulus contrast fixed at 98% Michelson).

#### Binocular rivalry experiments

In the motion rivalry experiments subjects had to fixate a 0.2° cross at the center of visual display throughout the trial. Shortly (1 sec) after the fixation cross appeared, RDKs with horizontal motion in the two opposite directions were presented to the left and right eye for 60 seconds. Thus, in each trial, signal dots either moved in the temporal-to-nasal or in the nasal-to-temporal direction. Motion directions were counterbalanced across the trials.

In all rivalry experiments subjects continuously reported their percepts by pressing and holding one of the two mouse buttons as long as either one of the two coherent motion percepts was dominant (exclusive percept). Both buttons had to be pressed if transparent or piecemeal percept occurred (mixed percept). If the stimuli did not elicit any mixed or coherent motion percept, that is if the subjects could not discern any visual motion, or if they perceived the stimuli as dynamic noise, no button had to be pressed (null percept).Stimuli for the left and right eye were manipulated in two different ways. In the *symmetric* condition, the changes in stimulus contrast/coherence were the same in the two eyes. In the *asymmetric* condition, stimulus contrast/coherence was kept fixed in one eye (contralateral eye), and varied from trial to trial in the other eye (ipsilateral eye).

#### Contrast manipulation

In the contrast experiments (5 subjects), the RDKs consisted of 100% coherently moving signal dots and we manipulated the dot luminance between 20 cd/m^2^ and 47 cd/m^2^ against a gray background of 15 cd/m^2^ (same background as in the 2AFC paradigm). This resulted in contrast levels in the range of 15 to 51% Michelson, with increments of about 1× the 75%-correct motion discrimination threshold for stimulus contrast (see above). Pilot experiments showed that motion rivalry stimuli presented at contrast levels around the subjects’ 75%-correct motion discrimination threshold (i.e., 6.4% Michelson) did not elicit any motion percept whatsoever. To make sure that our stimuli evoked a motion percept in all subjects under all conditions, the lowest contrast of the dots was therefore set to approximately 2× the average contrast motion discrimination threshold. The seven contrast levels were thus set to 15, 21, 27, 33, 39, 45 and 51% Michelson. The stimuli were presented in both symmetric and asymmetric conditions. For the asymmetric conditions, stimulus contrast in the contralateral eye was kept fixed at 33% Michelson (i.e., about 5× the contrast motion discrimination threshold).

#### Coherence manipulation

In the coherence experiments, the signal and noise dots were displayed at 14 cd/m^2^ against a black background of 0.12 cd/m^2^ (same background as in the 2AFC paradigm). This resulted in a fixed contrast of 98% Michelson across all trials. To match up with the contrast experiments, the lowest coherence value in the first coherence experiment (6 subjects) was set to approximately 2× the subjects’ average 75%-correct motion discrimination threshold for coherence (see above), and subsequent coherence levels were chosen at increments of about 1× the coherence motion discrimination threshold. Thus, the seven different coherence levels in this experiment were 9, 12, 16, 20, 23, 27 and 31%. As in the first contrast experiment, we applied both symmetric and asymmetric manipulations within each block of 38 trials. For the asymmetric rivalry conditions, motion coherence in the contralateral eye was kept fixed at 20% (i.e., about 5× the coherence motion discrimination threshold).

An additional coherence experiment (4 subjects) was performed to test the symmetric rivalry conditions for a wider range of coherence values comprising coherence levels of 2, 4, 6, 8, 12, 16, 20, 25, 30, 40, 50, 60, 80 and 100% (i.e., from 0.5 to 50× the average discrimination threshold).

Data were collected in blocks of 38 trials, except in the second coherence experiment, where each block consisted of 28 trials. Trials were presented in pseudorandom order with each stimulus configuration occurring once per block. For all experiments, each subject accomplished 6 blocks, which resulted in 12 trials per experimental condition after pooling the data from the two opponent motion-direction conditions (i.e., temporal-to-nasal and nasal-to-temporal).

### Data analysis

Based on the recorded button presses, we first marked all phases of exclusive leftward and rightward motion percepts as well as phases with mixed and/or `null’ percepts if present. Mixed percepts were determined from epochs where subjects pressed both buttons and null percepts from the intervals between two consecutive leftward, rightward or mixed motion percepts (i.e., epochs during which subjects pressed no button). Each particular state change of the buttons had to last at least 50 ms before it was counted as change in percept. This was done because subjects often had to operate both buttons to indicate a switch from one percept to another, resulting in almost simultaneous but slightly asynchronous presses/releases of the two buttons.

For each trial, we then calculated the mean duration of each percept state as well as its predominance, where predominance is defined as the percentage of the total viewing time during which a given state was dominant. Truncated percepts at the end of a trial were included in this predominance measure, but excluded from the computation of mean dominance durations. The resulting values were then averaged across trials, pooling coherent motion percepts according to the eye of origin (i.e., the ipsilateral and contralateral eye), and compared across conditions using repeated measures analysis of variance (ANOVA). We also fitted linear regression lines to the averaged data, and applied Student’s t-tests to evaluate systematic changes as a function of stimulus strength. Test results reported in the text always refer to the group statistics.

To account for the occurrence of non-exclusive percepts, we also quantified the changes in duration of the dominance alternation cycles (second coherence experiment). The duration of each cycle was taken from the onset of an exclusive dominance state (i.e., a coherent leftward/rightward motion percept) to the next-first onset of that same state following an epoch of exclusive dominance of the competing state (i.e., a coherent rightward/leftward motion percept), regardless of the presence/absence of any intervening mixed and/or null percepts.

**Figure 1:**
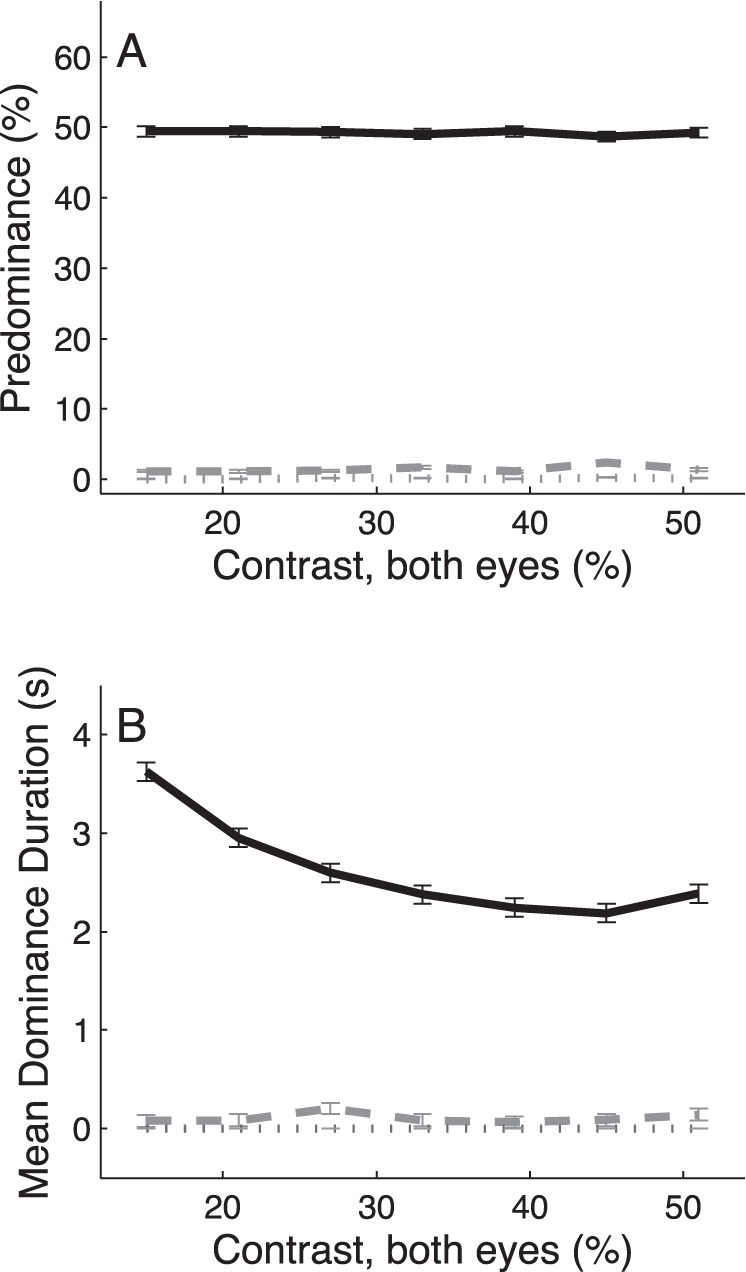
Contrast manipulation in both eyes. Predominance (A) and mean durations (B) of exclusive (black solid curves), mixed (gray dotted curves) and null (gray dashed curves) percepts as a function of stimulus contrast (in % Michelson) in the two eyes. Data are pooled across both eyes (exclusive percepts), and averaged across n = 5 subjects. Error bars indicate ±1 SEM as computed from the ANOVA sum of squares [47].

**Figure 2:**
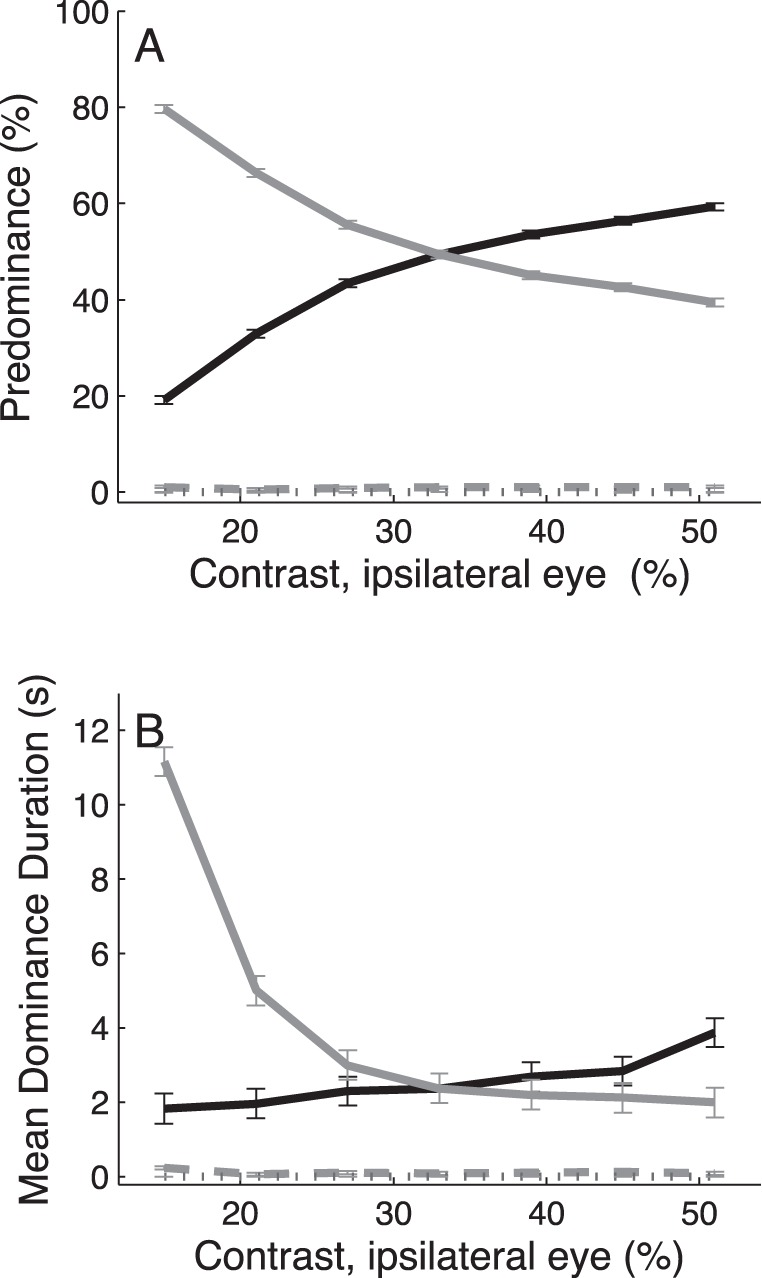
Contrast manipulation in one eye. Predominance (A) and mean dominance duration (B) of the ipsilateral (black solid curves) and contralateral (gray) eye both changed as a function of ipsilateral contrast. Mixed (gray dotted curves) and null percepts (gray dashed curves) remained almost absent. Data are averaged across n = 5 subjects. Error bars indicate ±1 SEM. Contrast of the coherently moving dots in the contralateral eye was fixed at 33% Michelson, which corresponded with 5× the subjects’ 75%-correct motion discrimination threshold for contrast (Methods).

**Figure 4:**
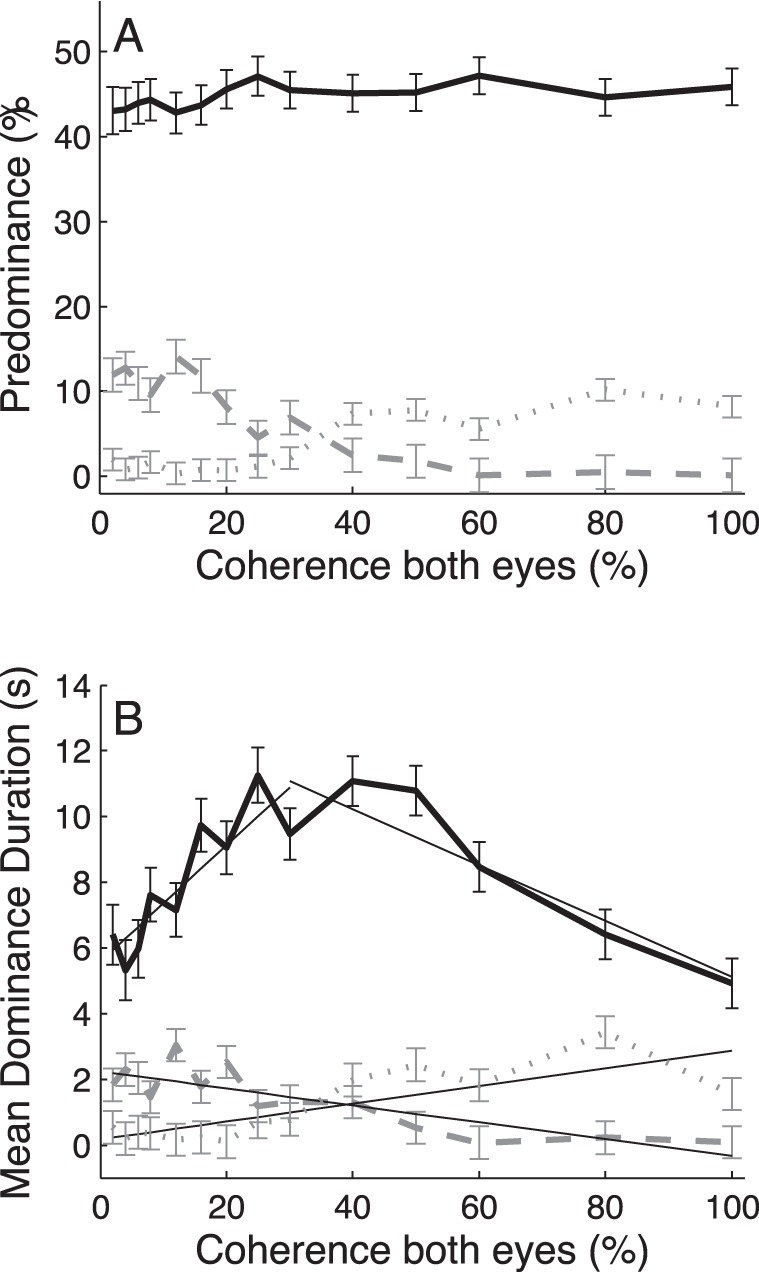
Coherence manipulation in both eyes. Predominance (A) and mean dominance durations (B) of exclusive (black solid curve), mixed (gray dotted curve) and null (gray dashed curve) percept as a function of motion coherence in the two eyes. Data averaged across n = 4 observers. Error bars indicate ±1 SEM. Gray line segments are linear regression lines fitted to sections of the data.

**Figure 5:**
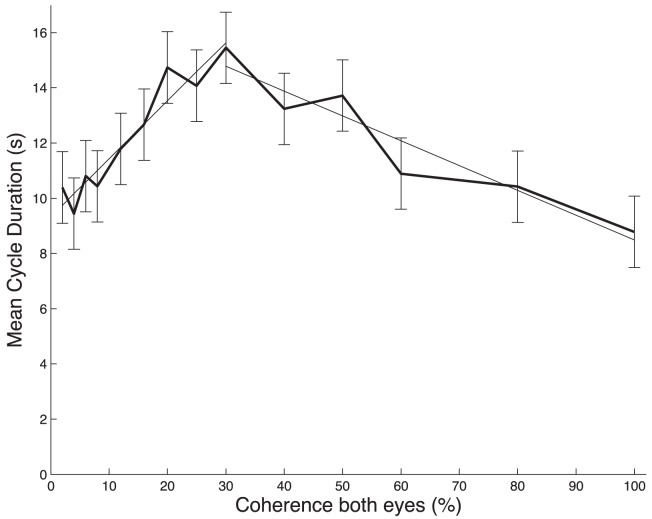
Mean durations of alternation cycles as a function of motion coherence in the symmetric rivalry conditions. Note that cycle durations first increased with increasing coherence up to ∼25% and then declined for higher motion coherence values. Data averaged across n = 4 observers. Error bars indicate ±1 SEM.

In some cases (Figs. 1B, 2, 4B and 5) the response curves were better described by quadratic or cubic polynomial functions (see Material S1). But since most of the variance in Figs. 1B and 2 was already explained by the linear terms, and since the inclusion of higher-order polynomial terms did not provide any further mechanistic insight, the main text only reports the results of our linear trend analyses. This first-order approximation only made our test statistics more conservative. To obtain a piece-wise linear approximation of the non-monotonic, n-shaped response functions in Figs. 4 and 5, we split them into two parts based on the location of their respective peaks. To obtain an objective estimate of the peak location, we determined the global maximum of the response curve from a cubic polynomial fit to the data.

## Results

### Symmetric Contrast Manipulations

We first analyzed the effect of manipulating stimulus contrast in the two eyes simultaneously. Note that there were no significant changes in predominance of the coherent motion percepts as a result of this symmetric contrast manipulation (Fig. 1A, solid curve; one-way ANOVA: F_6,829_ = 0.04, p>0.99). For all 5 subjects, average predominance of these exclusive motion percepts were close to 50% at all contrast levels, whereas the predominance of both mixed (dotted gray curve) and null (dashed gray curve) percepts was close to 0%. This indicates that decreasing the stimulus contrast down to 2× the motion discrimination threshold (Methods) did not limit the subjects’ ability to perceive coherent motion. Mean dominance durations (Fig. 1B), however, were significantly influenced by the contrast manipulations (one-way ANOVA: F_6,829_ = 29.89, p<<0.01). More specifically, in line with L4, we observed that the mean durations of their coherent motion percepts decreased significantly as a function of increasing stimulus contrast in the two eyes (mean ± SD slope of linear trend: α = −0.033±0.009; t-test, t_5_ = −3.57, p<0.02; see Material S1, for a more accurate description of this systematic decline using a 2^nd^ order polynomial fit). This behavior was observed in all 5 subjects (Material S1,Figs. S1 and S2).

### Asymmetric Contrast Manipulations

We then quantified how changing the contrast in one eye affected binocular rivalry. As shown in Figure 2, this manipulation evoked robustly different changes in exclusive dominance of the ipsilateral and contralateral eye (solid curves; two-way ANOVA, eye × contrast interaction, F_6,1662_ = 566.33, p<<0.01 for predominance and F_6,1651_ = 44.68, p<<0.01 for mean dominance duration) while the occurrence of mixed (dotted curves) and null percepts (dashed curves) remained close to zero. More specifically, in all our subjects, linear trend analysis of the coherent motion percepts showed that increasing the contrast in one eye, from levels below to levels above the fixed-eye contrast (33%), produced a significant increase in exclusive predominance of that same eye (slope of linear trend: α = 1.05±0.15, t-test, t_5_ = 6.88, p<<0.01), and a concomitant decrease in exclusive predominance of the other, contralateral eye (slope of linear trend: α = −1.06±0.15, t-test, t_5_ = −6.97, p<<0.01). Note, however, the changes in mean dominance duration of the manipulated eye were quite modest (slope of linear trend: α = 0.05±0.008, t-test, t_5_ = 5.77, p<0.01) compared with those of the contralateral eye (slope of linear trend: α = −0.20±0.07, t-test, t_5_ = −2.86, p<0.05); decreasing the contrast in one eye from 51% to 15% Michelson produced a robust increase in mean dominance durations of the contralateral eye. This response pattern, which was slightly better described with higher-order polynomials, was consistently observed in all 5 subjects (Figs. S3 and S4).

Although Fig. 2B shows that changes in one eye’s contrast mainly affected dominance durations of the higher contrast stimulus, as predicted by Brascamp’s revision of L2 [21], the effects of increasing contrast above the fixed-eye contrast (i.e., right-hand section of the curves) were relatively small in both eyes. To test if larger changes in dominance durations would occur for bigger increases in ipsilateral contrast, we conducted a second contrast experiment (Fig. S5) in which we tested contrasts between 51% and 83% Michelson. Our results indicated that for these higher contrasts, the mean dominance durations of the manipulated eye indeed increased further while the mean dominance durations of the contralateral eye remained largely unaffected.

### Asymmetric Coherence Manipulations

**Figure 3:**
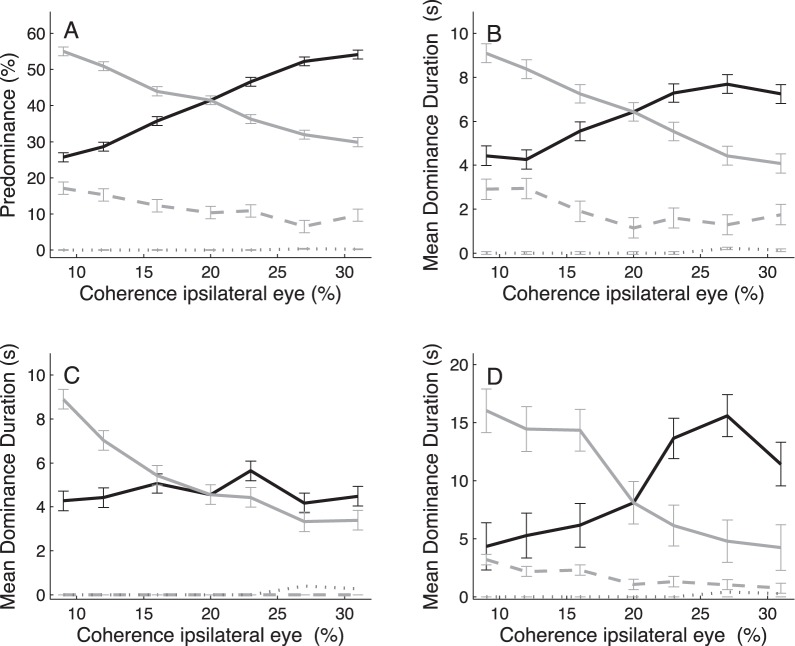
Motion coherence manipulation in one eye. A,B: Predominance (A) and mean dominance duration (B) of the ipsilateral (black solid curves) and contralateral (gray solid curves) eye both changed as a function of ipsilateral coherence. Mixed percepts (gray dotted curves) rarely occurred. Noise-like null percepts (gray dashed curves) comprised only ∼10% of the total viewing time, and their mean durations were comparatively short. Data averaged across n = 6 observers. C,D: Mean dominance durations from two individual subjects (S1 and S2) illustrating that observed response patterns ranged from asymmetric (C) to more or less mirror-symmetric (D). Coherence in the contralateral eye was fixed at 20%, which corresponded with 5× the subjects’ 75%-correct motion discrimination threshold for coherence (Methods). Error bars indicate ±1 SEM.

For the coherence experiments, we first quantified how binocular rivalry was influenced by changes in motion coherence in one eye while keeping it fixed in the other. Figure 3 shows the results from the first experiment in which the percentage of coherently moving dots ranged between 9 and 31%.

Note that changing the coherence in one eye led to significantly different changes in predominance (Fig. 3A, two-way ANOVA, eye × coherence interaction, F_6,1997_ = 122.99, p<<0.01) and mean dominance durations (Fig. 3B, two-way ANOVA, eye × coherence interaction, F_6,1952_ = 26.51, p<<0.01) of the ipsilateral and contralateral eye. Increasing the coherence in one eye, from levels below to levels above the fixed (20%) coherence in the other eye, caused a systematic increase in exclusive dominance of that same eye, both in terms of predominance (Fig. 3A, black curve slope of linear trend: α = 1.39±0.08, t-test, t_5_ = 18.43, p<<0.01) and mean dominance duration (Fig. 3B, black curve, slope of linear trend: α = 0.17±0.03, t-test, t_5_ = 5.89, p<0.01). These changes in exclusive dominance of the ipsilateral eye were accompanied by a systematic decrease in exclusive dominance of the contralateral eye. Both predominance (Fig. 3A, solid gray line, slope of linear trend: α = −1.17±0.07, t-test, t_5_ = −16.14, p<<0.01) and mean dominance duration (Fig. 3B, solid gray line, slope of linear trend: α = −0.24±0.01, t-test, t_5_ = −22.39, p<<0.01) of the contralateral eye decreased with increasing coherence in the manipulated eye. Note, however, that the overall changes in mean dominance duration resulted from significantly different response patterns across our subjects (multifactor ANOVA, subject × eye × coherence interaction: F_30,1887_ = 6.71, p<<0.01. see also Figs. S7 and S8, for individual subject data). At one end of the continuum (Fig. 3C), decreasing motion coherence in one eye mainly increased the dominance durations of the other eye in such a way that the dominance durations of that other eye ramped up as motion coherence in the manipulated eye decreased from levels above to levels below the fixed coherence in the contralateral eye. At the other end of the continuum (Fig. 3D), the effects of decreasing versus increasing coherence in one eye relative to the fixed coherence in the other eye (i.e., the left-hand and right-hand sections of the response curves, respectively) were much more symmetric; either manipulation strongly influences the dominance durations of both eyes in such a way that dominance durations of the eye receiving the strongest of the two stimuli increased while dominance durations of the other eye decreased, with the biggest changes occurring in the eye that received the stronger stimulus. These robust differences between subjects could not be accounted for by systematic differences in their non-exclusive percepts.

### Symmetric Coherence Manipulations

We then analyzed the effect of manipulating motion coherence in the two eyes simultaneously. For the first coherence experiment it appeared that the applied range of coherence values (9–31%) was too small to fully test the predictions of adaptation mutual-inhibition models against L4. We therefore performed a new experiment in which we applied symmetric coherence manipulations in the range of 2% to 100%. Figure 4 shows the outcomes of that second experiment. As before, the predominance (Fig. 4A) and mean dominance durations (Fig. 4B) of the exclusive (black solid curves), mixed (gray dash-dotted curves), and null (gray dashed curves) percepts are shown separately.

Note, that the symmetric coherence manipulations hardly influenced the predominance of the subjects’ coherent motion percepts (i.e., their reports of exclusive dominance of one of the two motion directions) (Fig. 4A, one-way ANOVA: F_13,1197_ = 0.83, p>0.6) while the mean durations of these percepts changed substantially (Fig. 4B, one-way ANOVA: F_13,1198_ = 6.86, p<<0.01). These changes were clearly not monotonically related to motion coherence; the mean durations of the coherent motion percepts showed first a robust increase as a function of increasing motion coherence for coherence values up to about 30% (as inferred from a cubic polynomial fit to the data; Methods) and then decreased gradually for higher motion coherence values. This response pattern was consistently observed in all 4 subjects (Figs. S9 and S10). Further post-hoc analysis with piece-wise linear regression indicated that mean dominance durations indeed increased significantly with increasing coherence (slope of trend line: α = 0.18±0.04, t-test, t_7_ = 4.69, p<0.01) in the lower (<30%) coherence range, and that they decreased significantly (slope of trend line: α = −0.09±0.02, t-test, t_4_ = −4.22, p<0.02) with increasing coherence in the higher (>30%) coherence range.

The significant increase in mean dominance durations with increasing coherence up to 30% is quite interesting, but due to the presence of non-exclusive percepts it is not immediately clear whether this result entails a violation of L4. The predominance of null percepts (gray dashed curves) indeed increased systematically with decreasing motion coherence and also their mean durations rose significantly (slope of trend line: α = −0.03±0.005, t-test, t_12_ = −5.34, p<<0.01). The latter might not be surprising given that the direction of coherent motion is more difficult to discern at lower coherence levels. Hence, one might suspect that the observed effect at low coherence values simply resulted from the fact that the coherent motion percepts gave way to noise-like null percepts. Note, however, that the changes in mean dominance duration were, on average, six times larger for the coherent motion percepts compared with those for the null percepts (i.e., slope of linear trend line α = 0.18±0.04 versus α = −0.03±0.005, respectively). Moreover, the increase in durations of the null-percepts as a function of decreasing coherence was paralleled by a significant decrease in durations of the mixed percept (Fig. 4B, gray dotted line, slope of linear trend: α = 0.03±0.006, t-test, t_12_ = 4.51, p<<0.01). Our findings thus point to a real increase in the duration of the alternation cycle with increasing stimulus strengths in the low coherence range, as predicted by adaptation mutual-inhibition models.

To test this notion, we also quantified the changes in mean duration of the dominance alternation cycles, where an individual alternation cycle was taken from the onset of an exclusive dominance state to the next-first onset of that same state occurring after exclusive dominance of the competing state (Methods). Figure 5 shows the changes in mean cycle duration as a function of coherence in the two eyes (one-way ANOVA: F_13,655_ = 2.56, p<0.01). Note, that the non-motonic n-shaped nature of this response curve is qualitatively very similar to the one found for mean dominance durations of the two exclusive motion percepts (c.f. Fig. 4B). This response pattern was consistently observed in all 4 subjects (Fig. S11). Importantly, the cycle durations indeed increased significantly with increasing coherence up to ∼30% (slope of trend line: α = 0.21±0.02, t-test, t_7_ = 8.78, p<0.01) and then decreased significantly for higher motion coherence values (slope of trend line: α = −0.09±0.01, t-test, t_4_ = −6.12, p<0.01).

## Discussion

In the present binocular rivalry experiments, we systematically varied the strength of visual motion stimuli in the two eyes by manipulating either the contrast or the coherence of the random-dot motion pattern in one eye or in both eyes simultaneously. In the asymmetric condition, we found that both contrast and coherence manipulations in one eye resulted in substantial dominance changes of both the ipsilateral and contralateral eye. The overall effect was that mean dominance durations of the eye receiving the stronger stimulus increased while mean dominance durations of the other eye decreased, albeit less steeply. But, where changes in dominance duration of the weaker stimulus were quite small in the contrast experiments, those changes were much larger in the coherence experiment, at least in some of our subjects. Furthermore, in all our subjects, the effects of increasing the contrast in one eye (relative to the other) were much weaker than the effect of decreasing the contrast in that same eye by a similar amount (in % Michelson), while these effects typically were more balanced in the coherence experiments. We also found striking differences between contrast and coherence manipulations in the symmetric condition. Increasing the contrast in the two eyes simultaneously produced a systematic, monotonic decrease in their mean dominance durations, but increases in coherence first increased and then decreased the mean dominance durations of both eyes. The same held true for durations of the alternation cycle, indicating a nonmonotonic change in the alternation rate. Our coherence experiments thus demonstrate clear violations of both L2 and L4. However, as we will discuss below, these violations are fully consistent with the predictions of adaptation mutual-inhibition models. Also the apparent discrepancy between the effects of contrast and coherence manipulations can be reconciled within this framework.

### Stimulus Strength

**Figure 6:**
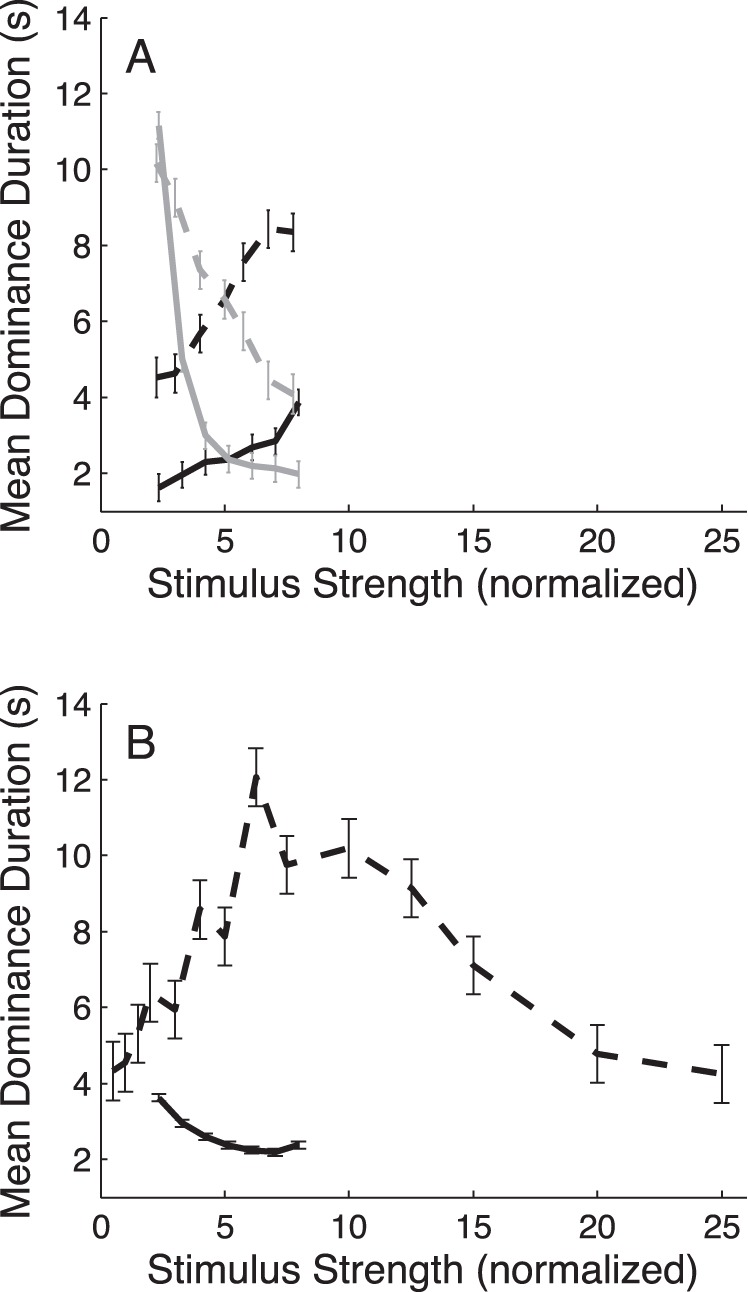
Comparison of contrast and coherence manipulations. Mean dominance durations of coherent motion percepts (i.e., exclusive dominance states) plotted as a function of stimulus strength, where stimulus strength was normalized by dividing the contrast and coherence values by their respective 75%-correct motion discrimination thresholds. (A) Mean dominance durations of the controlateral (gray) and ipsilateral (black) eye obtained with asymmetric contrast (solid curves) and coherence (dashed curves) manipulations. Data are from the contrast and first coherence experiments. (B) Mean dominance durations obtained with symmetric contrast and coherence manipulations. Circles and triangles represent averaged subject-data from the contrast and second coherence experiments, respectively. Error bars indicate ±1 SEM.

A direct comparison between the effects of contrast and coherence manipulations is not trivial because contrast and coherence define stimulus strength in different physical units. In an attempt to address this problem, we measured the subjects’ motion-direction discrimination thresholds for contrast and coherence, and normalized the stimulus strengths with respect to this psychophysical performance index. Although perception of a monocular image is clearly different when there is a rivaling stimulus in the other eye [39], such normalization might still provide a unified measure of stimulus strength for contrast and coherence. It appeared, however, that this linear rescaling did not work. This is shown in Fig. 6, where the mean dominance durations from the contrast (solid curves) and coherence (dashed curves) experiments are plotted as a function of normalized stimulus strength for unilateral (Fig. 6A) and bilateral (Fig 6B) stimulus manipulations (exclusive percepts only). The overall offset difference between the contrast and coherence response curves was in part due to the fact that data included different participants each having different mean dominance durations. Even so, it is clear that also the shapes of the contrast and coherence response curves are clearly different for both the symmetric and asymmetric conditions. For example, increasing the contrast in both eyes from 2 to 8 times the threshold produced a systematic decrease in mean dominance durations, while a decrease in mean dominance durations was only observed if the coherence increased beyond 6 times the threshold (Fig. 6B). Given that there may be complex interactions between contrast and motion sensitivity, it is possible that the differences between the contrast and coherence results are partly due to the fact that the fixed coherence in the contrast experiments (i.e., 100%) had a normalized stimulus strength of ∼25, whereas the fixed contrast in the coherence experiments (i.e., 98% Michelson) had a normalized strength of ∼15. The observed differences between the two sets of experiments could also arise from differences in nonlinear input scaling of contrast versus coherence. The simulations presented below indeed support this notion.

### Revised Version of L2

In the asymmetric condition, changes in mean dominance duration disobeyed L2. This was observed in the contrast experiments as well as the coherence experiments (Figs. 2 and 3). This result is consistent with the findings of Brascamp et al. [21] who demonstrated that, if tested for a wide range of stimulus contrasts, mean dominance durations of the contralateral and ipsilateral eye both undergo large-scale changes. In line with Brascamp’s revised L2, we found that increasing the stimulus strength in one eye had the biggest effect on the mean dominance durations of the stronger stimulus, except that this effect could be quite asymmetric. More specifically, increases in dominance durations of the stronger stimulus, which resulted from contrast decreases in the manipulated eye (i.e., in the other eye), were much steeper than those induced by contrast increases in that same eye. We could duplicate these results with static gratings similar to those used by Brascamp et al. [21] (see Fig. S6), indicating that this asymmetry is not unique for random-dot motion stimuli. For unilateral coherence manipulations, on the other hand, the results from our different subjects spanned a continuum, ranging from a quite asymmetric response pattern (Fig. 3C) qualitatively comparable to the one found for unilateral contrast manipulations, to a much more symmetric pattern with almost equally strong effects occurring for coherence increases versus coherence decreases (Fig. 3D). Furthermore, where contrast manipulations mainly influenced dominance durations of the stronger stimulus, coherence manipulations also had a substantial effect on dominance durations of the weaker stimulus, at least in some of our subjects.

### Model Simulations

**Figure 7:**
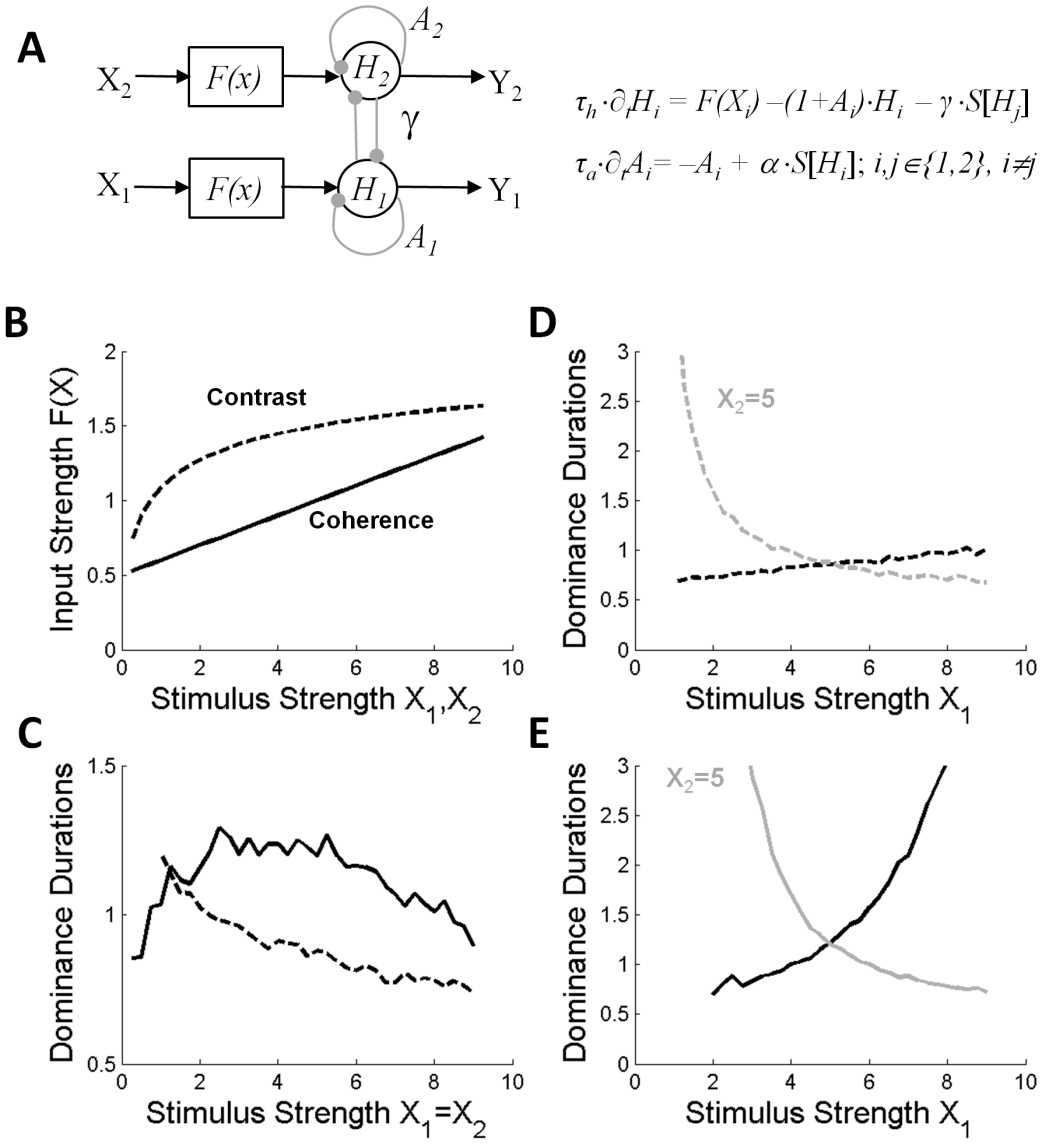
Adaptation mutual-inhibition models account for our results. (A) Model used in the simulations (modified after Noest et al., 2006). Each unit received visual input (X_i_) from one eye via a nonlinear input stage (F(x)). The dynamics of each unit were given by a set of differential equations which specified the ‘local field’ dynamics and the ‘shunting-type’ adaptation component of each unit. The local field activity of each unit (H_i_) was converted into a spike-rate output (Y_i_) via a sigmoid function (S(z) = z^2^/[z^2^+1] if z>0, otherwise S(z) = 0), and depended on the visual inputs (X_1_, X_2_), the adaptation dynamics (A_1_,A_2_), and the amount of cross inhibition (for details, see Noest et al., 2007). Parameters of the competition stage were: unit time constant, τ_h_ = 0.02s; adaptation time constant, τ_a_ = 4s, adaptation strength, α = 5; cross-inhibition gain, γ = 3.33 (adopted from Noest et al., 2006). Unit 1 and 2 were considered dominant if Y_1_>Y_2_ and Y_2_>Y_1_, respectively. (B) Different nonlinear input functions (F(x)) were used to simulate the effects of contrast and coherence manipulations. For contrast, we assumed a nonlinear compression function: F(x) = a⋅x^b^/[x^b^+1], with a = 2.17 and b = 0.5. For coherence, we assumed a linear relation with coherence plus a constant bias: F(x) = a⋅x+b, with a = 0.1 and b = 1. (C) Dominance durations of the two units when the two inputs were varied simultaneously (i.e, symmetric condition: X_1_ = X_2_). Input values are in arbitrary units. Same format as Figs. 1 and 4. Note peaked response curve for coherence and monotonic decrease for contrast. (D-E) Dominance durations of the two units as a function of X_1_ when X_2_ was kept constant (i.e., asymmetric condition, X_2_ = 5). Gray and black curves are the results for unit 1 and 2, respectively. Same format as Figs. 2 and 3. Note that mean dominance durations changed in both units but for ‘contrast’ manipulations (D), the biggest effect occurred in the contralateral unit (gray curve) while for ‘coherence’ manipulations (E) the effects were strongest in the unit which received the stronger input.

To better understand the apparent differences between the contrast and coherence experiments, we performed simulations with a simplified version of the bistable perception model by Noest et al. [4], which shares the basic properties of other adaptation mutual-inhibition models (Fig. 7A). Like most adaptation mutual-inhibition models of binocular rivalry, this model has two adapting units which cross-inhibit each other through their output. The cross-inhibition produces suppression of the initially weaker percept while the other one becomes dominant. The inhibitory influence of the dominant unit on the suppressed unit then slowly decays as a result of adaptation of the dominant unit, allowing the suppressed unit to (re)gain dominance. This, in turn, allows the previously-dominant unit to recover from adaptation, and restart the alternation cycle.

Simulations with this model indicated that the inter-subject differences observed in the coherence experiments, as well as the apparent difference between the contrast and coherence experiments, may be understood from differences in how the physical stimulus strengths map onto the neural inputs of the two competing populations. Figure 7B shows the two different mapping functions that we used. Each mapping function had only two free parameters. For contrast, we assumed a nonlinear compression function whereas for coherence we assumed linear modulation relative to a fixed baseline [as suggested by neurophysiologic data from, e.g., 40,41,42]. The parameters of these two functions were adjusted manually to roughly fit the model responses to our data while keeping all other model parameters fixed to their default value (after Noest et al., 2006). We thus had only two degrees of freedom to fit the shape of the response curves for the contrast and coherence experiments, respectively. Note that with these different functions we were indeed able to reproduce the qualitative differences that were observed between the coherence and contrast manipulations: For the symmetric conditions (Fig. 7C) we obtained a peaked and a monotonically decreasing response function, respectively. For the asymmetric conditions, the biggest effect occurred either in the contralateral unit (Fig. 7D) or in the unit which received the stronger input (Fig. 7E), depending on the nature of the input mapping function. This strongly suggests that contrast and coherence manipulations influence the competition in a similar fashion, albeit with different nonlinear scaling of the input. How the joint effect of contrast and coherence influences the rivalry dynamics remains an open question, which we did not address in this paper.

### Violation of L4

One intriguing feature of adaptation mutual-inhibition models is that increasing the two inputs *X*_1_ and *X*_2_ simultaneously yields an initial increase in percept durations followed by a decrease in percept durations for higher input strengths [26], [27]. The solid response curve in Fig. 7C (coherence) clearly illustrates this feature for the Noest model (Noest et al., 2007). This general inability of adaptation mutual-inhibition models to comply with L4 became a motivation for alternative approaches within a mutual inhibition framework [43] as well as models not based on adaptation [23]; [,28]. However, experimental data on L4 at low stimulus strengths are scarce. One reason for this is the limited ability of low contrast stimuli to evoke binocular rivalry [19], [29]. For example, orthogonal gratings dichoptically presented at low contrasts fuse into a plaid percept [29], [44]. Our subjects reported seeing no stimulus whatsoever once the contrast of the opponent motion stimuli in the two eyes dropped below 85% of the motion discrimination threshold for unambiguous stimuli (data not shown). Such low contrast values were therefore not included in the range of input strengths tested in our study. Within the range of contrasts that did elicit motion percepts, our results fully complied with L4, demonstrating no opposite trend in the lower contrast range (Fig. 1B).

Given the difficulty to elicit binocular rivalry at near-threshold contrasts, we tested L4 for low stimulus strengths by changing motion coherence instead of contrast. Note that decreasing coherence significantly below the motion discrimination threshold did not lead to a breakdown in bistable perception. Although subjects were given the instruction to press no button in case of having no coherent motion percept (null percept), or to press both buttons for a piecemeal or transparent percept (mixed percept), the predominance values indicated that either leftward or rightward motion percepts were dominant for nearly 80% of the time even if the coherence level was below the 75%-correct motion discrimination threshold (Fig. 4A). At these low coherence values, increases in stimulus strength did evoke robust increases in mean dominance durations (Fig. 4B), as predicted by adaptation mutual-inhibition models (Fig. 7C), and this increase continued for motion coherence values up to about 10× the subjects’ motion discrimination threshold.

The systematic increase in dominance of the noise percepts with decreasing coherence (Fig. 4) shows, that part of this effect could be due to the fact that the coherent percepts give way to noise-like percepts, which is not surprising, given that motion-direction discrimination becomes more difficult with decreasing motion coherence. However, we found clear evidence that task difficulty alone cannot explain why L4 was strongly violated by our data in the low coherence range. The decline in dominance durations of the coherent motion percepts with decreasing coherence was much bigger than the concomitant increase in dominance durations of the noise percepts. In addition, the latter was paralleled by a simultaneous decrease in dominance durations of the mixed percepts (Fig. 4B). Furthermore, our analysis of the alternation cycles, which accounted for the occurrence of mixed and noise percepts, clearly showed that the cycle durations decreased in the low coherence range (Fig. 5), as predicted by the model analysis of Shpiro [26]. We thus conclude that the observed violations of L4 are fully consistent with the predictions of adaptation mutual-inhibition models [26], [27].

### Alternative Representation of Motion Coherence?

As shown in Fig. 7, our data from both the contrast and coherence experiments are fully captured by adaptation mutual-inhibition models in which stimulus strength is represented by the amplitude of the inputs *X*_1_ and *X*_2_. In principle, however, our manipulations of motion coherence could instead have influenced the amount of noise in the populations (by default, in Fig. 7, the independent noise added to the units’ inputs was given by: *∂_t_N_i_ = −N_i/_τ_n_ +σ_i_⋅*√(*2/τ_n_*)*⋅η_i_* (*t*), with standard deviation *σ_i_* = 0.03 and timescale *τ_n_* = 0.1 s. *η_i_* (*t*) is white noise with zero mean and unit variance). We tested if this alternative possibility could also account for our data, but this was not the case (data not shown). When we fixed the noise *N_1_* added to the input of unit 1 (i.e., *I_1_ = F*(*X_1_*)*+N_1_* and manipulated the variance of the noise *N*_2_ added to the input of unit 2 to simulate the asymmetric condition (with mean input strengths fixed at *X*_1_ = *X*_2_ = 5), the mean dominance durations of *both* units decreased simultaneously and monotonically as a function of increasing noise *N*_2_. Such behavior is clearly different from both revised L2 and our data. Simulating the symmetric condition by simultaneously changing the amount of noise (*σ*_1_ = *σ*_2_) received by both neural populations, yielded results similar to the asymmetric condition, hence predicting solely a decrease in dominance durations as a function of increasing noise in both units. This is again different from both our data and L4.

### Non-exclusive Percepts

For simplicity, we did incorporate non-exclusive percepts in our model simulations. We note, however, that the observed increase in mixed motion percepts in the coherence experiments (Fig. 4) might be explained by the strength of those stimuli in relation to the strength of the cross-inhibition. The adaptation mutual-inhibition model of Fig. 7 indeed predicts that for sufficiently strong stimuli in both eyes the cross-inhibition is no longer able to fully suppress activity of either one of the two populations. This might have occurred for the higher coherent stimuli in the coherence experiments, because in those experiments the motion stimuli were also presented at comparatively high contrasts (i.e., 98% Michelson).

Thresholding the units’ activity levels could provide a means to introduce ‘null’ percepts, but we think a more elaborate model is called for. After all, there is a clear difference between low contrast stimuli which subjects cannot see all together, and noisy stimuli which subjects do see, but for which they cannot identify the direction of motion. Since motion-direction discrimination probably involves the integration of activity across populations of neurons broadly tuned to different directions of visual motion [e.g., 45,46], we speculate that binocular motion rivalry also involves competition between such populations of motion-sensitive neurons. Providing those populations with noisy inputs will result in flatter activation profiles, leading to longer and more often occurring epochs of perceptual uncertainty, while lowering the stimulus contrast will eventually elicit no response whatsoever.

### Conclusion

We found that L4, like L2, is only valid in a limited range of stimulus strengths. Outside that range, the opposite is true. These results support the validity of adaptation mutual-inhibition models for binocular rivalry both at low and at high stimulus strengths. The predictions of these models actually fit the experimental data obtained in our study better than Levelt’s classic L2 and L4 propositions do.

## Supporting Information

Figure S1 **Contrast manipulation in both eyes.** Top and bottom-left panels show predominance of exclusive (black solid curves), mixed (gray dotted curves) and null (gray dashed curves) percepts as a function of stimulus contrast (in % Michelson) in the two eyes from 5 individual subjects. In the bottom-right panel, solid lines are polynomial fits to the data (see Methods) for the exclusive (circles, α_0_ = 45.59±0.28 and α_1_ = −0.01±0.008), null (triangles, α_0_ = 0.85±0.48 and α_1_ = 0.02±0.01) and mixed (squares, α_0_ = −0.04±0.11 and α_1_ = 0.004±0.003) percept, averaged across n = 5 subjects. Error bars indicate ±1 SEM.
(EPS)

Figure S2 **Contrast manipulation in both eyes.** Top and bottom-left panels show mean dominance durations of exclusive (black solid curves), mixed (gray dotted curves) and null (gray dashed curves) percept as a function of stimulus contrast in the two eyes from 5 individual subjects. In the bottom-right panel, solid lines are polynomial fits to the data (see Methods) for the exclusive (circles, α_0_ = 5.60±0.16, α_1_ = −0.16±0.01 and α_2_ = 0.002±0.0002), null (triangles, α_0_ = 0.10±0.06 and α_1_ = 0.0003±0.002) and mixed (squares, α_0_ = 0.00±0.00 and α_1_x = 0.00±0.00) percept, averaged across n = 5 subjects. Error bars indicate ±1 SEM.
(EPS)

Figure S3 **Contrast manipulation in one eye.** Top and bottom-left panels show predominance of exclusive percept corresponding to the motion in the ipsilateral (black solid curves) and contralateral (gray solidcurves) eye, and predominance of mixed (gray dotted curves) and null (gray dashed curves) percept as a function of contrast in the ipsilateral eye, from 5 individual subjects. In the bottom-right panel, solid lines are polynomial fits to the averaged data from n = 5 subjects (see Methods) for the exclusive percept corresponding to the motion in the ipsilateral (black circles, α_0_ = −44.05±2.70, α_1_ = 5.78±0.28, α_2_ = −0.12±0.01 and α_3_ = −0.001±0.0001) and contralateral (gray circles, α_0_ = 140.48±4.00, α_1_ = −5.49±0.42, α_2_ = −0.11±0.01 and α_3_ = −0.0008±0.0001) eye, as well as for the null (triangles, α_0_ = 0.38±0.47 and α_1_ = 0.07±0.01) and mixed (squares, α_0_ = 0.00±0.00 and α_1_ = 0.00±0.00) percept. Error bars indicate ±1 SEM.
(EPS)

Figure S4 Contrast manipulation in one eye. Top and bottom-left panels show mean dominance durations of exclusive percept in the ipsilateral (black solid curves) and contralateral (gray solid curves) eye, and mean dominance durations of mixed (gray dotted curves) and null (gray dashed curves) percept as a function of contrast in the ipsilateral eye, from 5 individual subjects. In the bottom-right panel, solid lines are polynomial fits to the averaged data from n = 5 subjects (see Methods) for the exclusive percept corresponding to the motion in the ipsilateral (black circles, α_0_ = −0.93±0.30 and α_1_ = 0.05±0.01) and contralateral (gray circles, α_0_ = 42.88±3.98, α_1_ = −3.22±0.42, α_2_ = 0.08±0.01 and α_3_ = −0.0007±0.0001) eye, as well as for the null (triangles, α_0_ = 0.11±0.03 and α_1_ = 0.0004±0.0008) and mixed (squares, α_0_ = 0.00±0.00 and α_1_ = 0.00±0.00) percept. Error bars indicate ±1 SEM.
(EPS)

Figure S5 **Contrast manipulation in one eye.** Predominance (A) and mean dominance duration (B) of the ipsilateral (black) and contralateral (gray) eye both changed as a function of ipsilateral contrast. Solid lines represent data obtained in contrast experiment 1, averaged across the n = 6 participants (c.f., Fig. 2). Dashed lines show the results from contrast experiment 2 averaged across n = 3 subjects. Contrast of the coherently moving dots in the contralateral eye was fixed at 33% Michelson. Error bars indicate ±1 SEM.
(EPS)

Figure S6 **Static orthogonal gratings.** Predominance (A) and mean dominance duration (B) changed both in the ipsilateral (black) and contralateral (gray) eyes as a function of grating contrast in the ipsilateral eye. Contrast of the sinusoidal grating in the contralateral eye in was fixed at 33% Michelson. Data averaged across n = 5 subjects. Error bars indicate ±1 SEM.
(EPS)

Figure S7 **Coherence manipulation in one eye.** Top and bottom-left panels show predominance of exclusive percept in the ipsilateral (black solid curves) and contralateral (gray solid curves) eye, and predominance of mixed (gray dotted curves) and null (gray dashed curves) percept as a function of coherence in the ipsilateral eye. For the graphical purposes, the data from only n = 5 subjects are plotted. In the bottom-right panel, solid lines are polynomial fits to the averaged data from all n = 6 subjects (see Methods) for the exclusive percept corresponding to the motion in the ipsilateral (black circles, α_0_ = 13.24±1.59 and α_1_ = 1.39±0.08) and contralateral (gray circles, α_0_ = 64.40±1.53 and α_1_ = −1.17±0.07) eye, as well as for the null (triangles, α_0_ = 19.62±1.90 and α_1_ = −0.40±0.09) and mixed (squares, α_0_ = −0.21±0.13 and α_1_ = 0.02±0.006) percept. Error bars indicate ±1 SEM.
(EPS)

Figure S8 **Coherence manipulation in one eye.** Top and bottom-left panels show mean dominance durations of exclusive percept in the ipsilateral (black solid curves) and contralateral (gray solid curves) eye, and mean dominance durations of mixed (gray dotted curves) and null (gray dashed curves) percept as a function of coherence in the ipsilateral eye. For the graphical purposes, the data from only n = 5 subjects are plotted. In the bottom-right panel, solid lines are polynomial fits to the averaged data from all n = 6 subjects (see Methods) for the exclusive percept corresponding to the motion in the ipsilateral (black circles, α_0_ = 8.56±1.79, α_1_ = −0.99±0.31, α_2_ = 0.07±0.02 and α_3_ = −0.001±0.0003) and contralateral (gray circles, α_0_ = 11.18±0.23 and α_1_ = −0.24±0.01) eye, as well as for the null (triangles, α_0_ = 3.29±0.56 and α_1_ = −0.07±0.03) and mixed (squares, α_0_ = −0.11±0.07 and α_1_ = 0.008±0.003) percept. Error bars indicate ±1 SEM.
(EPS)

Figure S9 **Coherence manipulation in both eyes.** Top and bottom-left panels show predominance of exclusive (black solid curves), mixed (gray dotted curves) and null (gray dashed curves) percept as a function of stimulus coherence in the two eyes, from n = 4 individual subjects. In the bottom-right panel, solid lines are polynomial fits to the data (see Methods) for the exclusive (circles, α_0_ = 43.99±0.48 and α_1_ = −0.03±0.01), null (triangles, α_0_ = 11.71±1.00 and α_1_ = −0.15±0.02) and mixed (squares, α_0_ = 0.30±0.69 and α_1_ = 0.10±0.02) percept, averaged across n = 4 subjects. Error bars indicate ±1 SEM.
(EPS)

Figure S10 **Coherence manipulation in both eyes.** Top and bottom-left panels show mean dominance durations of exclusive (black solid curves), mixed (gray dotted curves) and null (gray dashed curves) percept as a function of stimulus coherence in the two eyes, from n = 4 individual subjects. In the bottom-right panel, solid lines are polynomial fits to the data (see Methods) for the exclusive (circles, α_0_ = 4.53±0.63, α_1_ = 0.39±0.07, α_2_ = −0.01±0.002 and α_3_ = (3.37±1.15)E10^−5^), null (triangles, α_0_ = 2.24±0.21 and α_1_ = −0.03±0.005) and mixed (squares, α_0_ = 0.18±0.26 and α_1_ = 0.03±0.006) percept, averaged across n = 4 subjects. Error bars indicate ±1 SEM.
(EPS)

Figure S11 **Coherence manipulation in both eyes.** Top and bottom-left panels show mean cycle durations of exclusive percept as a function of stimulus coherence in the two eyes, from n = 4 individual subjects. In the bottom-right panel, solid lines are polynomial fits to the data (see Methods) for the exclusive percept (α_0_ = 8.58±0.65, α_1_ = 0.41±0.07, α_2_ = −0.01±0.002 and α_3_ = (4.49±1.19)⋅10^−5^), averaged across n = 4 subjects. Error bars indicate ±1 SEM.
(EPS)

Material SI (DOCX)

## References

[1] Lehky SR (1988) An astable multivibrator model of binocular rivalry. Perception 17: 215–228. 3067209 10.1068/p170215

[2] Wilson HR (2003) Computational evidence for a rivalry hierarchy in vision. Proceedings of the National Academy of Sciences of the United States of America 100: 14499–14503. 14612564 10.1073/pnas.2333622100 PMC283620

[3] Freeman AW (2005) Multistage model for binocular rivalry. Journal of Neurophysiology 94: 4412–4420. 16148271 10.1152/jn.00557.2005

[4] Noest AJ, van Ee R, Nijs MM, van Wezel RJ (2007) Percept-choice sequences driven by interrupted ambiguous stimuli: a low-level neural model. Journal of Vision 7: 10. 10.1167/7.8.10 17685817

[5] Blake R (1989) A neural theory of binocular rivalry. Psychological Review 96: 145–167. 2648445 10.1037/0033-295x.96.1.145

[6] Moreno-Bote R, Rinzel J, Rubin N (2007) Noise-induced alternations in an attractor network model of perceptual bistability. Journal of Neurophysiology 98: 1125–1139. 17615138 10.1152/jn.00116.2007 PMC2702529

[7] Sundareswara R, Schrater PR (2008) Perceptual multistability predicted by search model for Bayesian decisions. Journal of Vision 8: 12 11–19. 10.1167/8.5.12 18842083

[8] Hohwy J, Roepstorff A, Friston K (2008) Predictive coding explains binocular rivalry: an epistemological review. Cognition 108: 687–701. 18649876 10.1016/j.cognition.2008.05.010

[9] Carter O, Cavanagh P (2007) Onset rivalry: brief presentation isolates an early independent phase of perceptual competition. PLoS ONE [Electronic Resource] 2: e343. 10.1371/journal.pone.0000343 PMC1828625 17406667

[10] van Ee R (2011) Percept-switch nucleation in binocular rivalry reveals local adaptation characteristics of early visual processing. Journal of Vision 11(2). 10.1167/11.2.13 21343327

[11] Blake R, Sobel KV, Gilroy LA (2003) Visual motion retards alternations between conflicting perceptual interpretations. Neuron 39: 869–878. 12948452 10.1016/s0896-6273(03)00495-1

[12] Kang MS, Blake R (2010) What causes alternations in dominance during binocular rivalry? Attention Perception & Psychophysics 72: 179–186. 10.3758/APP.72.1.179 PMC2811269 20045887

[13] Alais D, Cass J, O’Shea RP, Blake R (2010) Visual sensitivity underlying changes in visual consciousness. Current Biology 20: 1362–1367. 20598538 10.1016/j.cub.2010.06.015 PMC2918735

[14] Lankheet MJ (2006) Unraveling adaptation and mutual inhibition in perceptual rivalry. J Vis 6: 304–310. 16889470 10.1167/6.4.1

[15] Levelt WJM (1965) On binocular rivalry [Thesis, Leiden]. Te Assen,: Van Gorcum,. 110 p. p.

[16] Levelt WJ (1966) The alternation process in binocular rivalry. British Journal of Psychology 57: 225–238.

[17] Fox R, Rasche F (1969) Binocular rivalry and reciprocal inhibition. Perception & Psychophysics 5: 215–217.

[18] Blake R (1977) Threshold conditions for binocular rivalry. Journal of Experimental Psychology: Human Perception & Performance 3: 251–257. 864397 10.1037//0096-1523.3.2.251

[19] Bossink CJ, Stalmeier PF, De Weert CM (1993) A test of Levelt’s second proposition for binocular rivalry. Vision Research 33: 1413–1419. 8333162 10.1016/0042-6989(93)90047-z

[20] Mueller TJ, Blake R (1989) A fresh look at the temporal dynamics of binocular rivalry. Biological Cybernetics 61: 223–232. 2765591 10.1007/BF00198769

[21] Brascamp JW, van Ee R, Noest AJ, Jacobs RH, van den Berg AV (2006) The time course of binocular rivalry reveals a fundamental role of noise. Journal of Vision 6: 1244–1256. 17209732 10.1167/6.11.8

[22] Klink PC, van Ee R, van Wezel RJ (2008) General validity of Levelt’s propositions reveals common computational mechanisms for visual rivalry. PLoS ONE [Electronic Resource] 3: e3473. 10.1371/journal.pone.0003473 PMC2565840 18941522

[23] Moreno-Bote R, Shpiro A, Rinzel J, Rubin N (2010) Alternation rate in perceptual bistability is maximal at and symmetric around equi-dominance. Journal of Vision 10: 1. 10.1167/10.11.1 PMC3662020 20884496

[24] Laing CR, Chow CC (2002) A spiking neuron model for binocular rivalry. Journal of Computational Neuroscience 12: 39–53. 11932559 10.1023/a:1014942129705

[25] Wilson HR (2007) Minimal physiological conditions for binocular rivalry and rivalry memory. Vision Research 47: 2741–2750. 17764714 10.1016/j.visres.2007.07.007

[26] Shpiro A, Curtu R, Rinzel J, Rubin N (2007) Dynamical characteristics common to neuronal competition models. Journal of Neurophysiology 97: 462–473. 17065254 10.1152/jn.00604.2006 PMC2702527

[27] Curtu R, Shpiro A, Rubin N, Rinzel J (2008) Mechanisms for Frequency Control in Neuronal Competition Models. SIAM Journal Applied Dynamical Systems 7: 609–649. 10.1137/070705842 PMC2954747 20953287

[28] Ashwin P, Lavric A (2010) A low-dimensional model of binocular rivalry using winnerless competition. Physica D 239: 529–536.

[29] Liu L, Tyler CW, Schor CM (1992) Failure of rivalry at low contrast: Evidence of a suprathreshold binocular summation process. Vision Research 32: 1471–1479. 1455720 10.1016/0042-6989(92)90203-u

[30] Newsome WT, Britten KH, Movshon JA (1989) Neuronal correlates of a perceptual decision. Nature 341: 52–54. 2770878 10.1038/341052a0

[31] Britten KH, Shadlen MN, Newsome WT, Movshon JA (1992) The analysis of visual motion: a comparison of neuronal and psychophysical performance. Journal of Neuroscience 12: 4745–4765. 1464765 10.1523/JNEUROSCI.12-12-04745.1992 PMC6575768

[32] Britten KH, Newsome WT, Shadlen MN, Celebrini S, Movshon JA (1996) A relationship between behavioral choice and the visual responses of neurons in macaque MT. Visual Neuroscience 13: 87–100. 8730992 10.1017/s095252380000715x

[33] Shadlen MN, Newsome WT (2001) Neural basis of perceptual decisions in the parietal cortex (area LIP) of the rhesus monkey. Journal of Neurophysiology 86: 1916–1936. 11600651 10.1152/jn.2001.86.4.1916

[34] Roitman JD, Shadlen MN (2002) Response of neurons in the lateral intraparietal area during a combined visual discrimination reaction time task. Journal of Neuroscience 22: 9475–9489. 12417672 10.1523/JNEUROSCI.22-21-09475.2002 PMC6758024

[35] Gold JI, Shadlen MN (2002) Banburismus and the brain: decoding the relationship between sensory stimuli, decisions, and reward. Neuron 36: 299–308. 12383783 10.1016/s0896-6273(02)00971-6

[36] Platonov A, Goossens J (2010) The role of the multisensory integration in binocular rivalry dynamics FENS Forum Abstract 139.17.

[37] Brainard DH (1997) The Psychophysics Toolbox. Spatial Vision 10: 433–436. 9176952

[38] Pelli DG (1997) The VideoToolbox software for visual psychophysics: Transforming numbers into movies. Spatial Vision 10: 437–442. 9176953

[39] Andrews TJ, Blakemore C (1999) Form and motion have independent access to consciousness. Nature Neuroscience 2: 405–406. 10321242 10.1038/8068

[40] Sclar G, Maunsell JH, Lennie P (1990) Coding of image contrast in central visual pathways of the macaque monkey. Vision Res 30: 1–10. 2321355 10.1016/0042-6989(90)90123-3

[41] Albrecht DG, Hamilton DB (1982) Striate Cortex of Monkey and Cat - Contrast Response Function. Journal of Neurophysiology 48: 217–237. 7119846 10.1152/jn.1982.48.1.217

[42] Britten KH, Shadlen MN, Newsome WT, Movshon JA (1993) Responses of neurons in macaque MT to stochastic motion signals. Vis Neurosci 10: 1157–1169. 8257671 10.1017/s0952523800010269

[43] Seely J, Chow CC (2011) Role of mutual inhibition in binocular rivalry. Journal of Neurophysiology 106: 2136–2150. 21775721 10.1152/jn.00228.2011 PMC3296268

[44] Burke D, Alais D, Wenderoth P (1999) Determinants of fusion of dichoptically presented orthogonal gratings. Perception 28: 73–88. 10627854 10.1068/p2694

[45] Shadlen MN, Britten KH, Newsome WT, Movshon JA (1996) A computational analysis of the relationship between neuronal and behavioral responses to visual motion. Journal of Neuroscience 16: 1486–1510. 8778300 10.1523/JNEUROSCI.16-04-01486.1996 PMC6578557

[46] Jazayeri M, Movshon JA (2006) Optimal representation of sensory information by neural populations. Nature Neuroscience 9: 690–696. 16617339 10.1038/nn1691

[47] Loftus GR, Masson MEJ (1994) Using Confidence Intervals in Within-Subject Designs. Psychonomic Bulletin & Review 1: 476–490. 24203555 10.3758/BF03210951

