# Supplementary material for: Influence of Contrast and Coherence on the Temporal Dynamics of Binocular Motion Rivalry
Source: PLoS One. 2013 Aug 14;8(8):e71931. doi: 10.1371/journal.pone.0071931 (PMC3743782; doi:10.1371/journal.pone.0071931)
Supplement: Material SI — (DOCX) [file pone.0071931.s012.docx]

**Influence of Contrast and Coherence on the temporal dynamics of binocular motion rivalry**

Artem Platonov and Jeroen Goossens*

* Radboud University Nijmegen Medical Centre, Donders Institute for Brain, Cognition and Behavior, Department of Cognitive Neuroscience, Section Biophysics.

***Supplementary material***

**Methods**

The data from contrast experiment 1 and coherence experiment 1 and 2 were averaged across trials for each individual subject, compared across conditions and plotted as a function of stimulus strength. The results were further averaged across subjects and fitted with polynomial regression lines (i.e., *y=α0+α1x+α2x2…+αnxn*). We tested each individual regression coefficient with a t-test and applied forward variable selection to decide the appropriate degree of the polynomial regression model. Higher-order terms were included only if they reached statistical significance at p<0.05. Error bars in the graphs indicate ±1 SEM as computed from the ANOVA sum of squares ([Loftus & Masson, 1994](#_ENREF_30)).

**First contrast experiment, results from individual subjects**

**--- Supplementary Figure 1 in File S1 ---**

**--- Supplementary Figure 2 in File S2 ---**

**--- Supplementary Figure 3 in File S3 ---**

**--- Supplementary Figure 4 in File S4 ---**

**Second contrast experiment**

To test if larger changes in dominance durations would occur for higher increases in ipsilateral contrast, we conducted a second contrast experiment with 3 subjects in which contrast was further augmented in the ipsilateral eye to 51% and 83% Michelson, while keeping the contralateral contrast fixed at 33%.

**Method**

To test the asymmetric conditions for a wider range of contrast levels, we augmented the monitor’s luminance. This allowed us to set the luminance of stimuli in the ipsilateral eye at 47 cd/m2, 83 cd/m2 and 162 cd/m2 against a gray background of 15 cd/m2. This resulted in three additional contrast levels: 51, 70 and 83% while keeping the stimulus contrast in the contralateral eye fixed at 33%. Otherwise, the setup was the same, and subjects had the same instructions as in the first contrast experiment.

**--- Supplementary Figure 5 in File S5 ---**

**Results**

Figure S5 plots the averaged results of the second contrast experiment (dashed curves) together with the outcomes from the first contrast experiment (solid curves) for predominance (Fig. S5A) and mean dominance durations (Fig. S5B). The data from this additional experiment shows that for the higher contrast values, the mean dominance durations of the ipsilateral eye indeed increased (slope of trend line: α=0.10±0.01, t-tests, p<0.05) while the mean dominance durations of the contralateral eye remained largely unaffected (slope of trend line: α=-0.009±0.007, t-tests, p>0.05). Thus, the overall effect was that changes in one eye’s contrast mainly affected dominance durations of the higher contrast stimulus as predicted by Braskcamp’s revised version of L2 ([Brascamp et al., 2006](#_ENREF_6)).

**Static control experiment**

To determine if the behavior obtained with our random-dot motion stimuli can be compared with findings already established for static stimuli, we conducted a control experiment with 5 subjects in which we used static orthogonal gratings similar to those used by Brascamp et al. (2006), testing the same contrast levels as in the first motion contrast experiment.

**Method**

In this control experiment two orthogonal gratings with a diameter of 4^o^ and an average luminance of 15 cd/m^2^ for both stimulus and background were used. The spatial frequency of these gratings was 1.5 cycles/deg. To support binocular alignment, both images were encircled by a ring with four additional line segments extending outward in the cardinal directions. The tilt of each grating was 45^o^ from the vertical axis. In a given trial, gratings could be tilted either inwards or outwards. The number of trials with inward and outward tilts was counterbalanced across trials.

**--- Supplementary Figure 6 in File S6 ---**

**Results**

The averaged results are plotted in Fig. S6. Both predominance (Fig. S6A) and mean dominance durations (Fig. S6B) changed systematically as a function of contrast in the manipulated eye. The changes in binocular rivalry dynamics closely matched the changes that we observed in the first contrast experiment of the main study (c.f., Fig. 2). In line with Brascamp’s revised L2, increasing the stimulus strength in one eye mainly affected the mean dominance durations of the stronger stimulus (Fig. S6B). However, as in the first motion contrast experiment of the main study, the effects of increasing the contrast in one eye (re. to the other) were much weaker than the effect of decreasing the contrast in that same eye by a similar amount.

**First coherence experiment, results from individual subjects**

**--- Supplementary Figure 7 in File S7 ---**

**--- Supplementary Figure 8 in File S8 ---**

**Second coherence experiment, results from individual subjects**

**--- Supplementary Figure 9 in File S9 ---**

**--- Supplementary Figure 10 in File S10 ---**

**--- Supplementary Figure 11 in File S11 ---**
